# Multi-Fault Detection of Rolling Element Bearings under Harsh Working Condition Using IMF-Based Adaptive Envelope Order Analysis

**DOI:** 10.3390/s141120320

**Published:** 2014-10-28

**Authors:** Ming Zhao, Jing Lin, Xiaoqiang Xu, Xuejun Li

**Affiliations:** 1 School of Mechanical Engineering, Xi'an Jiaotong University, Xi'an 710049, China; E-Mails: zhaomingxjtu@mail.xjtu.edu.cn (M.Z.); xuxiaoqiang@stu.xjtu.edu.cn (X.X.); 2 State Key Laboratory for Manufacturing Systems Engineering, Xi'an Jiaotong University, Xi'an 710049, China; 3 College of Mechanical and Electrical Engineering, Hunan University of Science and Technology, Xiangtan 411201, China; E-Mail: hnkjdxlxj@163.com

**Keywords:** rolling element bearing, multi-fault diagnosis, time-varying rotating speed, fault sensitive matrix

## Abstract

When operating under harsh condition (e.g., time-varying speed and load, large shocks), the vibration signals of rolling element bearings are always manifested as low signal noise ratio, non-stationary statistical parameters, which cause difficulties for current diagnostic methods. As such, an IMF-based adaptive envelope order analysis (IMF-AEOA) is proposed for bearing fault detection under such conditions. This approach is established through combining the ensemble empirical mode decomposition (EEMD), envelope order tracking and fault sensitive analysis. In this scheme, EEMD provides an effective way to adaptively decompose the raw vibration signal into IMFs with different frequency bands. The envelope order tracking is further employed to transform the envelope of each IMF to angular domain to eliminate the spectral smearing induced by speed variation, which makes the bearing characteristic frequencies more clear and discernible in the envelope order spectrum. Finally, a fault sensitive matrix is established to select the optimal IMF containing the richest diagnostic information for final decision making. The effectiveness of IMF-AEOA is validated by simulated signal and experimental data from locomotive bearings. The result shows that IMF-AEOA could accurately identify both single and multiple faults of bearing even under time-varying rotating speed and large extraneous shocks.

## 1. Introduction

Rolling element bearings are common and vital mechanical components that are widely used in rotating machinery, and their malfunction may lead to uncomfortable vibration and noise, or even breakdown of the entire production line. As a result, it is crucial to detect bearing faults before a catastrophic failure occurs. A variety of monitoring and diagnostic techniques for rolling element bearings have been developed in recent years. Among those, vibration analysis has been proven to be the most fundamental and versatile one that plays an important role in bearing faults diagnosis.

When the rolling element passes through a faulty surface, a force impact is generated and this in turn excites the resonance frequency of the bearing system [1,2]. As the bearing rotates, these impulses will occur periodically with a frequency uniquely determined by the location of the defect. For this reason, the detection of faults in bearings is commonly achieved by identifying the bearing characteristic frequencies (BCFs), *i.e.*, ball pass frequency of inner-race (BPFI), ball pass frequency of outer-race (BPFO), or ball spin frequency (BSF). However, those impulses are very weak at the early stage of faults and are usually overwhelmed by measurement noise and other vibration sources (such as gear mesh and rotor unbalance), which causes difficulties for the early fault detection of bearings. For the purpose of signal denoising and weak signature enhancement, a variety of signal processing techniques have been proposed in recent years. They mainly include high frequency resonance technique (HFR) [3], spectral kurtosis [4–6], wavelet analysis [7,8], empirical mode decomposition [9,10], cyclostationary approach [11–15], minimum entropy deconvolution [16,17] and stochastic resonance [18–21] *etc.* Among those, the HFR is the most widely used since it could extract the fault-related amplitude-modulation that resides in a narrow band around the resonance and may minimize the effects of interfering signals in lower frequency range. Nevertheless, the major challenge in the application of HFR lies in how to choose the optimal frequency band for demodulation [22]. In early days, the demodulation frequency band was selected either by trial and error, or by shock testing using a hammer, both of which are rather inconvenient for practical applications. To address this issue, the spectral kurtosis (SK) method was intensively investigated by Antoni and Randall [4,5]. It is shown that SK is an effective statistical tool which can indicate not only transient components in the signal but also their locations in the frequency domain, thus providing a guideline for the optimal demodulation bandwidth selection in the envelope analysis. However, the original SK is time-consuming and not suitable for on-line fault detection. To overcome this issue, a 1/3-binary tree fast kurtogram estimator was further proposed Antoni, which made on-line condition monitoring a reality [6]. Since then, improvements of both SK and the kurtogram have attracted increasingly attention of researchers [23]. By introducing Wavelet Package Transform (WPT), Lei *et al.* [24] presented an improved kurtogram method which employs WPT as the filter-bank to overcome the shortcomings of the original kurtogram. A novel tool called Protrugram [25] is proposed by Barszcz and Jabłoński in 2011 for the optimal band selection, which is proven to be more effective than kurtogram in detecting the transient under heavy noise. In the same year, Wang *et al.* [22] proposed an adaptive spectral kurtosis for the multiple bearing faults detection. Considering the low time-frequency resolution of traditional kurtogram, Li *et al.* [26] developed a continuous-scale mathematical morphology (CSMM) scheme to locate the optimal scale band that best reflects the impulsive feature for more reliable fault signature extraction. Inspired by wavelet packet transform, an enhanced kurtogram was recently presented by Wang *et al.* [27]. In that method, the kurtosis values are calculated based on the envelope signals extracted from wavelet packet nodes at different depths, instead of band-pass filtered waveforms.

Although the aforementioned methods have shown different levels of success in bearing fault diagnosis, it is noted that most of those methods assume that the frequency band with largest kurtosis value is the resonance excited by the fault, and regard it as the best choice for demodulation. However, this assumption is not valid in many practical applications, especially when the bearing runs under harsh operation conditions. For instance, the measured signals of on-line locomotive bearing monitoring system are always corrupted by the impacts when the wheel goes through the joints between two successive railway tracks; the bearing signal of high-speed spindle usually suffered from transient vibration due to the non-continuous milling process. In addition, some incidental spikes caused by electromagnetic interference are frequently encountered in industrial applications. All the interferences mentioned above are impulse-like, which are very similar to the signature caused by bearing fault. Moreover, those interferences are always characterized by high amplitude and short-duration, thus have even larger kurtosis values than those of fault signatures. For this reason, when the maximum-kurtosis based approaches are applied to those signals, the strong interference will probably be extracted, however leaving the real fault signature undetected.

Besides, the traditional diagnostic methods are also premised on the assumption that the bearing is operating at a constant speed [28]. However, there are a variety of applications where the bearings experience different extents of speed variations due to non-stationary working condition. For instance, the rotating speed (RS) of automobile bearings are varying with its driving speed, the RS of mining excavator bearings are affected by the external load [29], and the RS of a wind turbine main bearing fluctuates with the wind power and speed [30]. In those cases, the repetition frequencies of impacts are also time-varying, hence the impulse signal and its envelope are non-stationary in nature. The direct application of frequency-based methods to those signals will result in spectral smearing and false diagnosis, even though the fault-induced impulses are extracted effectively [31,32].

This paper aims to provide a reliable and effective bearing diagnostic method capable of extracting fault signatures from the strong impulse interferences. In addition, it is also expected that this method applies not only to bearings under constant speed, but also those under variable speeds. For this purpose, an enhanced bearing signal analysis technique called IMF-AEOA is proposed by integrating the individual merits of ensemble empirical mode decomposition (EEMD), envelope order tracking and fault sensitive analysis. Compared with WT and HFR, the decomposition process of EEMD is data-driven and self-adaptive according to the natural oscillatory mode embedded in the signal, thus providing a fine separation of fault-related signal from the background noise and other interference. In addition, since the envelope of each IMF is resampled from time domain to angular domain, the spectral smearing problem encountered in varying speed cases is addressed successfully. By performing envelope order analysis (EOA), the BCFs of real faults are enhanced in the order domain due to angular cyclostationary characteristics, while the interferences are weakened since they are randomly distributed in the vibration signal. On this basis, a fault sensitive matrix (FSM) is further established to quantitatively assess how much diagnostic information each IMF has, with which the optimal component containing the richest diagnostic information may be identified for final decision making. By combining those advantages of EEMD, EOA and FSM, both single and multi-fault of bearing can be detected effectively even under varying speed operations and strong interferences.

The rest of this article is organized as follows: After a briefly review of EMD and EEMD in Section 2, the principle and implementation of proposed bearing diagnostic technique, IMF-AEOA, is elaborated in Section 3. The advantages of IMF-AEOA in interference resistance, multi-fault identification as well as variable speed application are demonstrated by simulation analysis in Section 4. The effectiveness of this approach is validated by application to locomotive bearing diagnosis in Section 5. Finally, some conclusions are drawn in Section 6.

## 2. A Briefly Review of EMD and EEMD

Before the introduction of EEMD, we will first give a briefly review of the classical Empirical Mode Decomposition (EMD) technique. EMD is a novel and effective signal processing technique, originally proposed by Huang *et al.* [33] in 1998. It can decompose a complicated signal into a set of complete and almost orthogonal components called intrinsic mode functions (IMFs), and each IMF represents one simple oscillatory signal mode [10]. It has been proven to be quite versatile for extracting signals from data generated in noisy nonlinear and non-stationary processes. In comparison with the WT, the decomposition process of EMD is data-driven and self-adaptive, according to the natural oscillatory mode embedded in the signal, rather than the pre-determined mother function. Those properties make the EMD a powerful tool in a wide variety of applications, such as voice recognition, system identification, medicine and biology, system control, *etc*. Studies on EMD applied to fault diagnosis of rotating machinery have also grown at a very rapid rate in the past few years, covering the diagnostic problems of rotors, gears and bearings.

However, the original EMD algorithm is not perfect. One of the major drawbacks of EMD is the mode mixing problem [34], which not only leads to serious aliasing in time-frequency distributions, but also makes the physical meanings of individual IMFs unclear. To alleviate this problem, a noise-assisted data analysis method, called ensemble empirical mode decomposition, was proposed by Wu and Huang [35] in 2009. The EEMD method first adds white noise with finite amplitude to the analyzed signals. Since the white noise is uniformly distributed throughout the frequency domain, no missing scales are present and hence the components in different scales of the signal are automatically projected onto proper scales of reference established by the white noise in the background [35]. As a result, the mode mixing due to the existence of intermittency is overcome effectively. Finally, the added white noise can be decreased or even completely canceled out in the ensemble mean of sufficient trails. In contrast to EMD, EEMD has better scale separation ability and more robust to mode mixing problem, which make it an appropriate candidate for analyzing the bearing vibration signals that inevitably contain intensive noises and other interference.

Based on the above discussion, the algorithm of EEMD can be summarized as follows:

(1) Initialize the number of trials in the ensemble, *J*, and the amplitude of added white noise. Set trial number *j* = 1;
(2) Generate a white noise series with the preset amplitude and add it to the analyzed signal, *i.e.*:
  (1) $$x_{j}(t)=x(t)+n_{j}(t)$$
  where *n_j_*(*t*) is the *j*-th added noise signal, *x_j_*(*t*) denotes the noise-added signal of the *j*-th tril.
(3) Decompose the white noise-added signal into *M* IMFs by EMD, and we can obtain:
  (2) $$x_{j}(t)=\overset{M}{\underset{m=1}{\text{∑}}}c_{j,m}(t)+r_{j,M}(t)$$
  where *c_j,m_*(*t*) and *r_j,M_*(*t*) demote the *m*th IMF and the residue of the *j*-th trial, respectively; *M* is the number of IMF of the *j*-th trial.
(4) If the trial number is smaller than the termination number, *i.e.*, *j* < *J*, then go to step (2) with *j* = *j* + 1. Repeat steps (2) and (3) again, but with independent random white noise signals each time.
(5) Calculate the ensemble mean of corresponding IMFs in each trail as the final result:
  (3) $${\bar{c}}_{m}(t)=\frac{1}{J}\sum_{j=1}^{J}c_{j,m}(t),m=1,2,\cdots M.$$

Generally, the noise amplitude is selected about 0.2–0.3 times the standard deviation of the original signal. However, since we primarily concern the high-frequency resonances where the bearing fault signatures residing in, the noise amplitude may be selected much smaller so as to separate those high-frequency components better as suggested in [35,36]. For this reason, the noise amplitude is set as 0.1 times the standard deviation of the original signal in our practice. Under this amplitude, an ensemble number of 100 is sufficient to suppress the added noise. Using such a signal processing technique, a complicated non-stationary bearing signal, *x*(*t*), could finally decomposed into a finite number of IMFs representing the natural oscillatory mode of *x*(*t*). For more details about EEMD readers may refer to [35].

## 3. The Proposed IMF-AEOA Method for Bearing Fault Diagnosis

**Figure 1.**
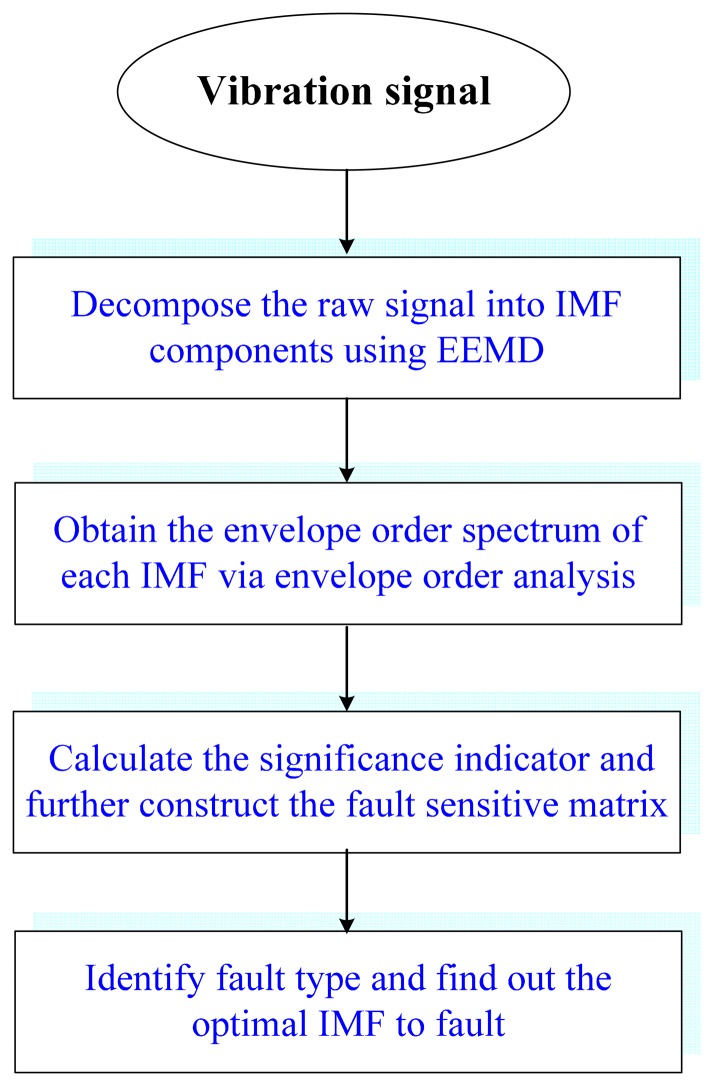
Flowchart of proposed IMF-AEOA.

**Figure 2.**
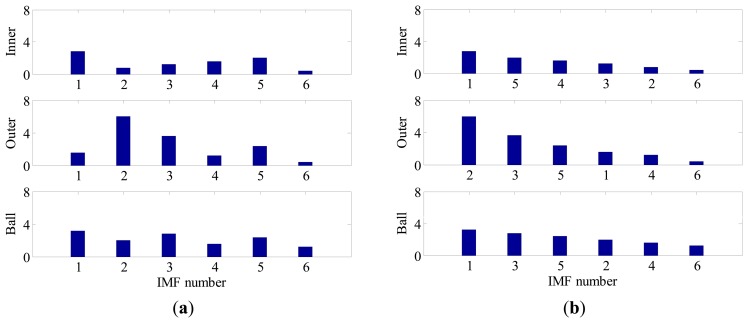
The bar plot of FSM: (**a**) before sorting; (**b**) after sorting.

To effectively detect the faults of rolling element bearings under harsh working condition, an IMF-based adaptive envelope order analysis (IMF-AEOA) technique is proposed in this section. The flow chart of the IMF-AEOA is shown in Figure 1, and its principle and implementation are elaborated as follows:

Step 1 Decompose the Raw Vibration Signal into IMFs Using EEMD
  As discussed previously, the vibration signal of bearings in industrial applications is rather complicated, and always contains impulses caused by bearing faults, the rotating harmonics due to unbalance and misalignment, the strong meshing vibration coming from gears, and the high-amplitude impulses resulting from other interference, *etc.* Therefore, it is advisable to separate those components before more advanced signal processing techniques are applied. To achieve this, EEMD is utilized to decompose the raw vibration into different subbands in terms of IMFs. Each IMF contains signal components within certain oscillation frequency range. Since the signal decomposition scheme is data-driven and self-adaptive, the EEMD can provide a nature separation of signal components according to their oscillation characteristics.
Step 2 Perform Envelope Order Analysis on Each IMF
  After applying the EEMD to the bearing signal, the impulses related to bearing faults may be decomposed into certain IMFs. Nevertheless, for incipient bearing faults, the signatures may be so weak that we can hardly identify them in the waveform of each IMF. Although the envelope spectrum is an effective tool for bearing fault diagnosis, however, the envelope of impulses is non-stationary under speed-varying working conditions, which in turn leads to the smearing effect in the envelope spectrum. Therefore, the envelope spectrum cannot be used directly for those signals. To overcome this limitation, the envelope order analysis (EOA) is performed on each IMF. In this step, the envelope of each IMF, *ĉ_i_*(*t*), is first obtained by calculating the modulus of its analytic signal, which is given by:
  (4) $${\hat{c}}_{i}(t)=c_{i}(t)+j\text{H}\left[c_{i}(t)\right]$$
  where H denotes the Hilbert transform.
  Then the envelopes are resampled with equal angular increments according to the instantaneous speed *v*(*t*) of bearing shaft. In practice, *v*(*t*) can be calculated either from a tachometer signal or the vibration signal itself [37]. Through the above procedure, the non-stationary envelope signal in time-domain can be transformed into a cyclostationary one in the angular domain. By performing Fourier transform on the resampled signals, we can get the envelope order spectrums of each IMF. The BCFs of real faults are enhanced in the envelope order spectrum due to their inherent angular cyclostationary characteristics, in the meantime the strong interferences are eliminated since they are randomly distributed in the vibration signal.
Step 3 Find out the Optimal IMFs Using Fault Sensitive Matrix
  For the signal acquired from a complex mechanical system, the number of decomposed IMFs can reach up to a dozen or more. It is a boring and time-consuming work to diagnose the fault by checking each IMF via visual inspection. In order for this method to be applicable in an on-line monitoring system, the health status or the fault types of bearing should be identified in an automatic manner. Moreover, since the fault-induced impact may simultaneously excite several resonance zones of the bearing system, the diagnostic information may distribute in multiple frequency bands. Therefore, several interesting and crucial problems arise in bearing fault diagnosis: How to find the most sensitive IMF to certain faults? Does the diagnostic information of different faults reside in the same IMF? In previous research works, the most sensitive IMF is usually selected according to its kurtosis value [10]. As pointed out previously, the IMF with largest kurtosis value is probably caused by interference, rather than fault signatures.
  To address this issue, an automatic optimal IMF selection scheme based on fault sensitive matrix (FSM) is established. In this scheme, a significance indicator (SI) for fault signature, *S_m,type_* is first proposed to measure the sensitivity of *m*-th IMF to a certain type of fault. Taking inner-race fault for example, the SI is defined as the energy ratio between the spectral component at BPFI and the local noise level around it, which is given as follows:
  (5) $$S_{m,\text{Inner}}=\frac{A_{m}(f_{\text{BPFI}})}{\text{RMS}{\left[A_{m}(f)\right]}_{f\in u}},u=\left[0.5f_{\text{BPFI}},1.5f_{\text{BPFI}}\right]$$
  where *A_m_*(*f_BPFI_*) denotes the amplitude of BPFI in the envelope order spectrum of *m*-th IMF, while the denominator in Equation (5) is the root mean square (RMS) value of the local background noise in the range of 0.5*f_BPFI_* ∼1.5*f_BPFI_*.
  If there is no inner-race fault, the SI will remain a low value since no BPFI component is provoked in the envelope order spectrum. However, if an inner-race fault occurs, those indicators will increase to different degrees for different IMFs. Therefore, the sensitivity of each IMF to inner-race fault could be evaluated according to its SI, and the optimal one may be determined. From the definition in Equation (5), we can see that the SI can also be extend to outer-race and rolling element faults directly. By taking all the possible faults into consideration, a fault sensitive matrix of dimension 3 × *M* can be constructed as follows:
  (6) $$\left[\begin{array}{cccc} S_{1,\text{Inner}} & S_{2,\text{Inner}} & \cdots & S_{M,\text{Inner}}\\ S_{1,\text{Outer}} & S_{2,\text{Outer}} & \cdots & S_{M,\text{Outer}}\\ S_{1,\text{Ball}} & S_{2,\text{Ball}} & \cdots & S_{M,\text{Ball}} \end{array}\right]$$
  Each row of the matrix reveals the sensitivities of different IMFs to a certain fault, while each column gives the sensitivities of a certain IMF to different faults. Figure 2a illustrates the corresponding bar plot of FSM. For better interpretation, the SIs in each row are sorted in a descend order as shown in Figure 2b. By using such a representation, one can comprehensively discover the relevance between IMFs and bearing fault signature, from high to low.
Step 4 Identify the Fault Type of Bearing
  From the above property of FSM, it is rational to conclude that the existence and the severity of bearing faults could also be determined if an appropriate threshold alarm is imposed on each FSM row. In practice, how to select the threshold value is an important issue. A small threshold may lead to false alarms, while a big threshold may lead to missing alarms. In this paper, the threshold value used is 4. It was determined based on the consideration that, for a non-fault bearing signal, the vibration waveform is generally dominated by Gaussian distributed white noise whose spectrum is almost flat in the frequency domain, hence the risk that a spectrum line exceeding four times the mean energy of its neighborhood is rather small. However, when a fault occurs the amplitude of BCF increases dramatically and can easily reach this threshold according to our experimental and in-suit bearing signal analysis. By impose the above threshold to each row of FSM, the health condition of bearing can be identified. The merit of this diagnostic scheme is that it not only applies to single fault, but also suitable for multi-fault cases.

## 4. Simulation Analysis

Generally, the vibration signal of a faulty rolling element bearing is mainly composed of three parts as given in Equation (7) [28]. The first part represents a series of impulses excited by defect, where *A_i_* is the amplitude of the *i*th impulse, *T_i_* is the time of its occurrence. The second part denotes the vibration harmonics caused by bearing imbalance or gear meshing. In this term, *B_n_* and *β_n_* are the amplitude and initial phase of the *n*th harmonic, *f*(*t*) is the instantaneous rotating frequency of bearing shaft. The third part *n*(*t*) stands for the white noise in the measurement:

(7) $$x(t)=\sum_{i}A_{i}s(t-T_{i})+\sum_{n}B_{n}\cos\left(2\pi nf(t)+\beta_{n}\right)+n(t)$$

In this simulation, a bearing with fixed outer-race and rotating inner-race is investigated. It's assumed that a local defect occurs on outer-race, whose characteristic frequency (*i.e.*, BPFO) is 6 times the rotating frequency of shaft. The fault excited bearing vibration is simulated by an exponentially decaying sinusoid with the following form:

(8) $$s(t)=e^{-\alpha t}\sin(2\pi f_{r}t)$$

where *f_r_* is the resonance frequency of the bearing system, which takes a values of 2000 Hz here, *α* is the damping ratio of the impulse, which takes 500 Hz here. The gear vibration is simulated as the 16*th* rotating harmonic of shaft, and its amplitude and phase are *B*_16_ = 0.4, *β*_16_ = π/6, respectively. The sampling frequency and time length are specified as 20,000 Hz and 1 s.

### 4.1. Application to Bearing Diagnosis with Varying Speed and Random Impulsive Noise

**Figure 3.**
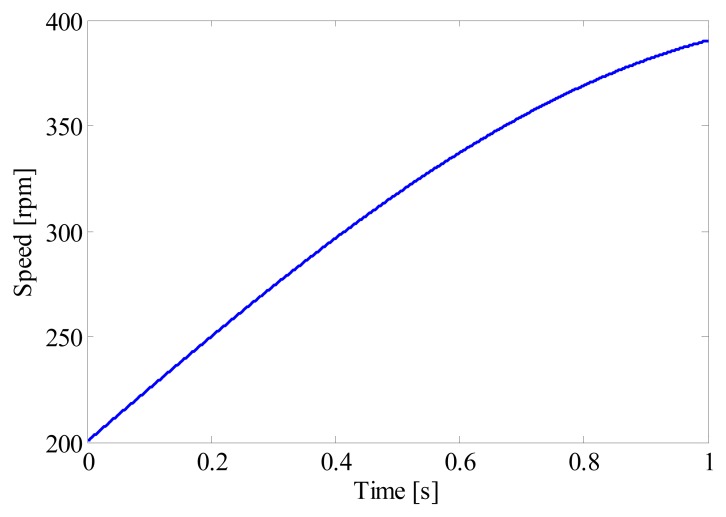
The speed curve of a bearing.

Bearings served in the industrial field usually suffer harsh working conditions. In order to establish a more realistic model, two frequently encountered scenarios are taken into account in this simulation, *i.e.*, time-varying rotating speed and random impulsive noise. To investigate the influence of speed variation on the performance of proposed method, a non-linear run up process is simulated by Equation (9), the speed curve of which is given in Figure 3:

(9) f(t)=[200+200⋅sin(2π⋅0.2⋅t)]/60

**Figure 4.**
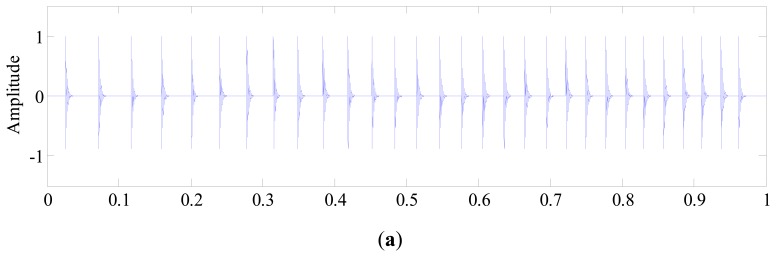

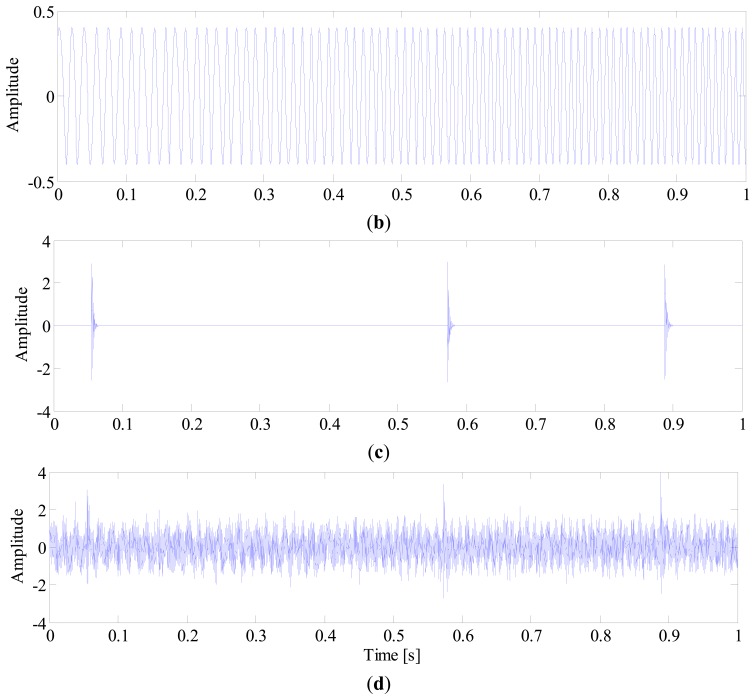
The simulated bearing vibration signal: (**a**) fault impulses *p*(*t*); (**b**) gear vibration *g*(*t*); (**c**) extraneous interferences *e*(*t*); (**d**) noisy compound signal *x*(*t*).

Figure 4a,b show the simulated fault impulses *p*(*t*) and gear mesh vibration *g*(*t*), respectively. It is noted that the impulses are not equally spaced in the time domain due to speed variation, and the gear mesh period becomes shorter as the rotation speed increases. To simulate the extraneous interferences encountered in real-case applications, several random amplitude and short duration bursts, *e*(*t*), with center frequency of 6000 Hz are generated and plotted in Figure 4c. By adding Gaussian distributed white noise, a noisy compound signal *x*(*t*) with SNR of −3 dB is finally obtained as illustrated in Figure 4d. From this figure, the impulses generated by fault can hardly be observed due to the heavy noise.

**Figure 5.**
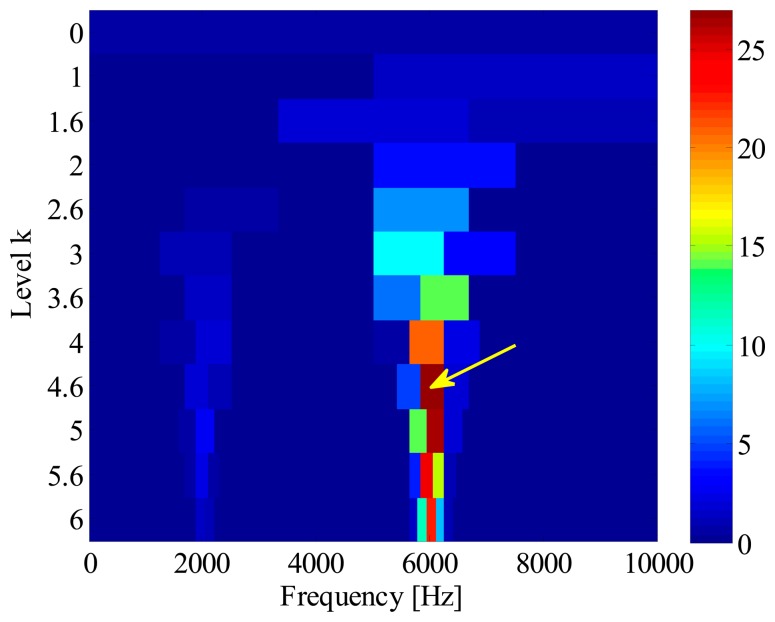
Kurtogram of simulated out-race fault signal.

**Figure 6.**
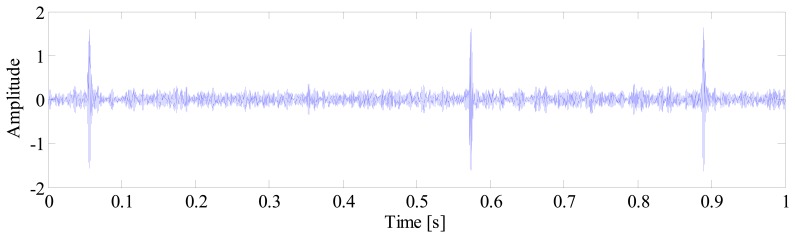
The band-pass filtered signal.

In order to extract the fault feature, a SK analysis method based on fast-kurtogram [6] is applied to the simulation signal. This signal is decomposed into six frequency levels, with a 1/3-binary tree structure. The corresponding kurtogram is presented in Figure 5, from which a kurtosis dominant frequency-band with centre frequency of 6041.7 Hz and bandwidth of 416.7 Hz is clearly identified. With this information, an optimal band-pass filter is further designed to extract the impulses from the raw signal. Figure 6 illustrates the filtered signal. However, it is surprising to observe that the extracted signals actually come from the interference as illustrated in Figure 4c, rather than the fault-induced impulses as we expected. The reason for this failure diagnosis lies in the fact that the conventional SK assumes that the frequency band with the largest kurtosis value is the frequency region where the fault impulses lie. Obviously, this assumption does not hold in the situations where strong non-Gaussian interferences exist. In fact, kurtosis is only a measure of the peakiness of a signal, but without any source identification. The more sparse distribution and sharper peaks a signal contains, the larger kurtosis value it has. Taking this simulation for instance, due to sporadic distribution and short duration, the kurtosis value of interference *e*(*t*) can reach up to 251, while the kurtosis value of the fault signal *p*(*t*) is only 25. It explains why the fault impulses are ignored when SK is applied to the simulated signal.

**Figure 7.**
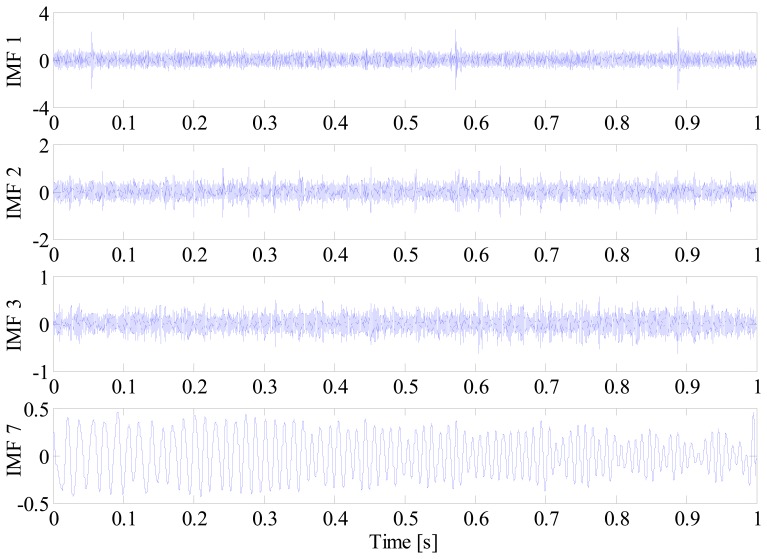
Several representative IMFs obtained by EEMD.

On the other hand, to demonstrate the effectiveness of proposed method, the IMF-AEOA is applied to the same signal by the following procedures. Firstly, the raw signal is decomposed into IMFs by EEMD. For conciseness, only several representative IMFs out of 11 IMFs are presented in Figure 7. It can be seen from this figure that the EEMD provides a natural and adaptive decomposition of the raw signal. The signal components with different oscillation characteristics are decomposed into different IMFs. For instance, the strong interferences become visible in IMF1, the fault-induced impulses, to some extent, can be observed in IMF2, while the rotating harmonics of bearing are mainly located in higher order IMFs with relatively low oscillation frequency, e.g., the IMF7. However, due to the heavy noise and time-varying signal structure, it is still impossible to determine the fault type by measuring the time-interval between two successive impulses in time domain, which is frequently employed in literatures. For this reason, more effective post-processing techniques are required.

**Figure 8.**
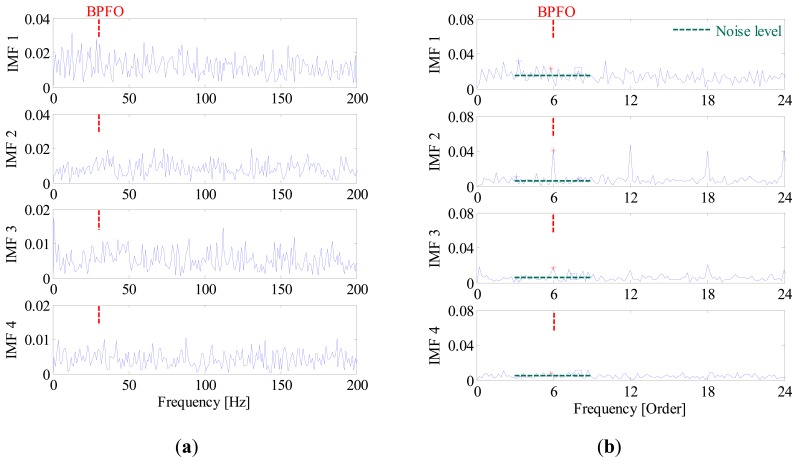
Comparison between (**a**) envelope spectrum and (**b**) envelope order spectrum for IMFs.

As we know, envelope spectral analysis is only applicable to the diagnosis of bearings working under stationary conditions. When ESA is applied to the bearing vibration signal or its IMFs under varying speed, a smearing phenomenon will occur as shown in Figure 8a, from which the BPFO cannot be identified in any IMF due to this smearing. To overcome this issue, envelope order analysis is utilized to process the IMFs in the proposed method. Since the envelope signal is resampled with equal increments in the angular domain, the frequency modulation effect caused by speed variation is removed effectively. For comparison, the envelope order spectrum (EOS) of each IMF is shown in Figure 8b. From the EOS of the IMF2, it is observed that the BPFO and harmonics of real fault are enhanced in the order domain due to its angular cyclostationary characteristics. At the same time, since strong interferences are randomly distributed in the vibration signal, their influence on the bearing diagnosis is eliminated completely in the EOS.

**Figure 9.**
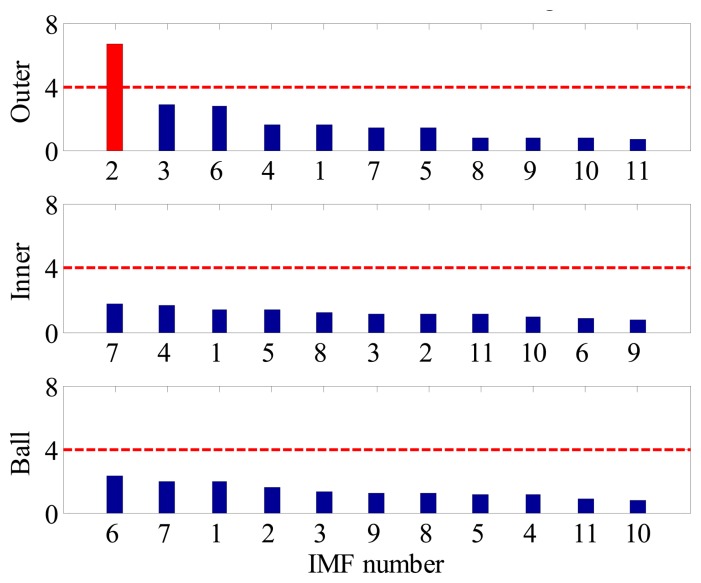
FSM of the simulated signal.

Although the fault type of bearings can be identified by visual inspection of BCFs in each EOS, such a diagnostic manner is inconvenient and time consuming. Moreover, for a data-driven signal decomposition approach, it is also desirable to establish a quantitative assessment of how much diagnostic information one IMF has. For the above purposes, the SI of each IMF to different type of fault is calculated according to Section 3. Figure 9 shows the FSM of the simulated signal. It is noted that the IMF2 is most sensitive to outer-race faults, and the corresponding SI is far above the threshold line. This signature clear indicates that an outer-race fault has occurs. On the other hand, since the SIs corresponding to inner-race and ball faults are all below the threshold line, so one can conclude that the inner-race and rolling elements are in a healthy state.

### 4.2. Case 2: Application in Multi-Fault Detection of Bearings

**Figure 10.**
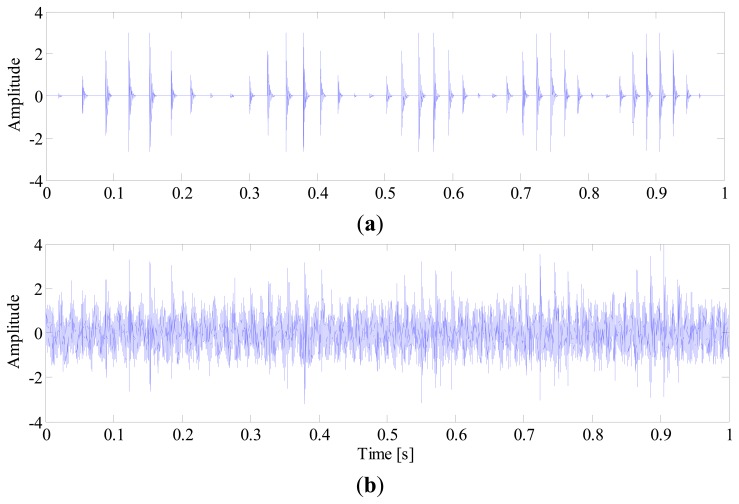
(**a**) Simulated inner-race fault signal; (**b**) compound signal.

**Figure 11.**
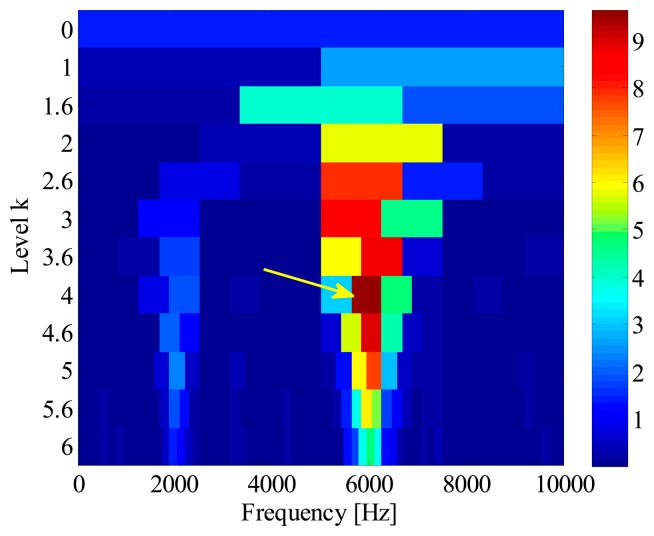
Kurtogram of simulated multi-fault signal.

**Figure12.**
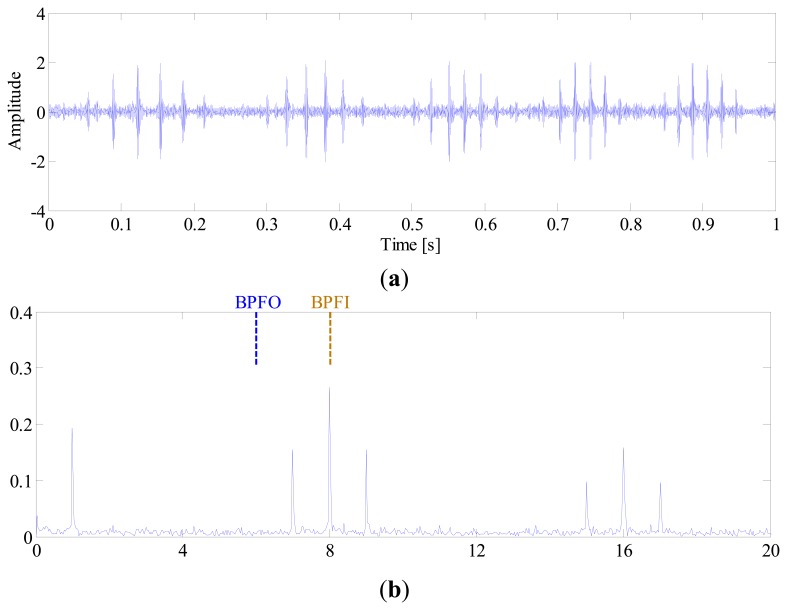
(**a**) Waveform and (**b**) envelope order spectrum of band-pass filtered signal.

Once a bearing defect occurs, the local stress increases significantly as the rolling element passed by. As a consequence, a single bearing fault will develop into a multi-fault in a very short time due to high stress. Another advantage of the proposed method is its ability for multi-fault detection. To demonstrate this, an inner-race fault signal is generated as shown in Figure 10a. Different from an outer-race fault, the energy of measured vibration signal of an inner-race fault is influenced by the load zone and time-varying transmission path. In this work, those effects are simulated by imposing an amplitude modulation function *a*(*t*) to the impulse train, which is given by:

(10) a(t)=[1−cos(θ(t))]/2

where *θ*(*t*) denotes the instantaneous angular position of the inner-race fault on the bearing. Since different faults may excite different frequency bands of the system, the resonance frequency of an inner-race fault is specified as 6000 Hz in this simulation. Figure 10b illustrates the final compound signal, which is generated by adding the inner-race fault to the previous signal *x*(*t*) as given in Figure 4d. By applying SK to the simulated signal, a frequency band with a center frequency of 5937.5 Hz and bandwidth of 625 Hz is identified in the kurtogram as presented in Figure 11. Figure 12a shows the band-pass filtered signal, from which the impulses induced by the inner-race fault can be identified. Figure 12b shows the envelope order spectrum of the filtered signal, from which one can only identify the BPFI, while leaving the outer-race fault undetected. This drawback is due to the fact that the conventional SK only focuses on the sole frequency-band with the largest kurtosis value. Therefore, in the case of multi-faults, the bearing defects with relative smaller kurtosis value may be ignored.

**Figure13.**
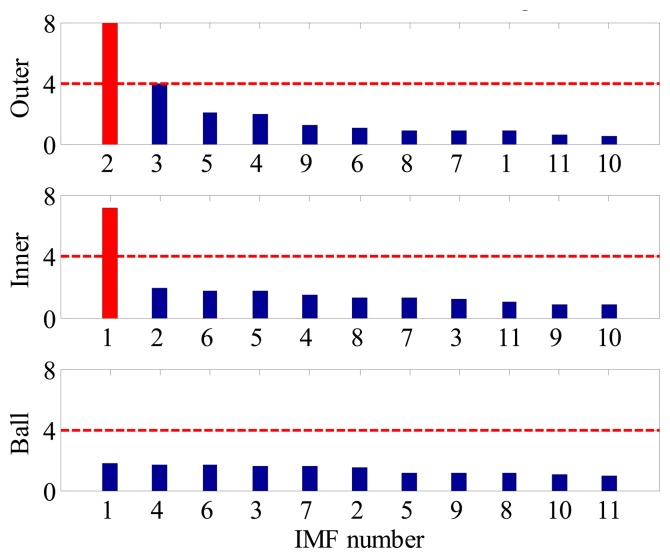
FSM of simulated multi-fault signal.

**Figure 14.**
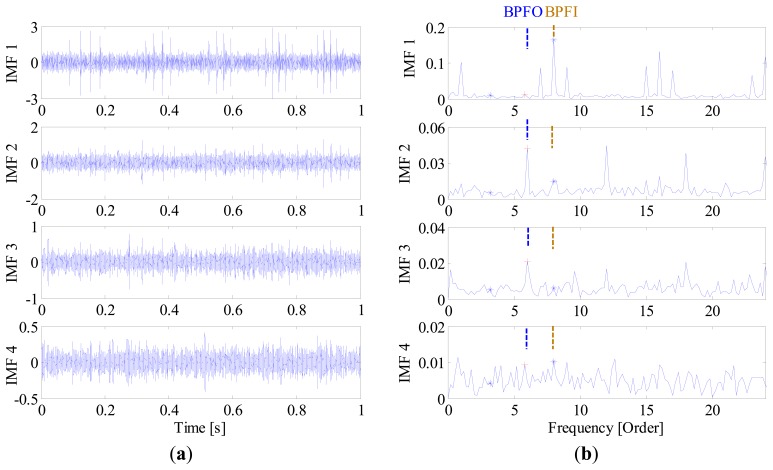
(**a**) Waveforms and (**b**) envelope order spectrums of the first 4 IMF components.

In contrast, since all the decomposed frequency bands are equally treated before envelope order analysis, the limitation mentioned above can be overcome effectively in the proposed method. Figure 13 presents the FSM of the simulated signal, from which the inner-race fault and outer-race faults can be identified clearly. Moreover, it is interesting to find in FSM that the IMF2 is most sensitive one to the outer-race fault, while IMF1 is the most sensitive one to the inner-race fault. To further validate the effectiveness of FSM, the waveforms and EOS of the first four IMFs are plotted in Figure 14a,b, respectively. It is obvious that the BPFI and its modulation sidebands are clearly revealed in the EOS of IMF1. Similarly, the diagnostic information of outer race fault mainly resides in the IMF2.

## 5. Application to Fault Diagnosis of Locomotive Bearings

**Figure 15.**
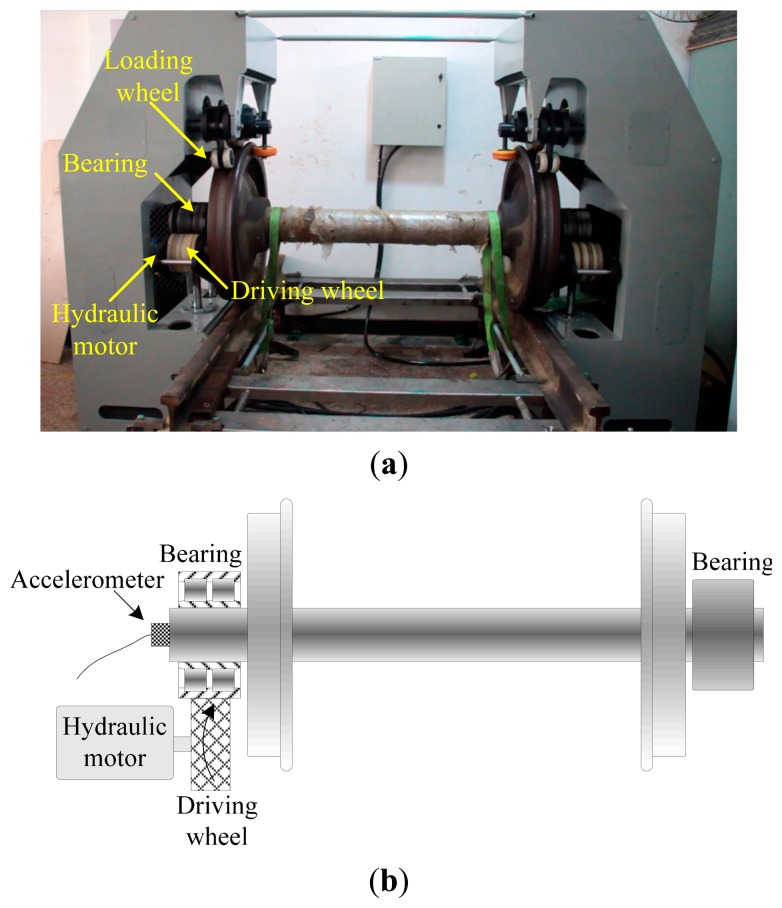
(**a**) The locomotive bearing test bench; (**b**) its schematic view.

To validate its effectiveness, the proposed method is applied to the fault detection of locomotive bearings in this section. The locomotive bearing test bench and its schematic are presented in Figure 15a,b, respectively. The outer-race of the bearing is driven by a nylon driving wheel connected to a hydraulic motor, while the inner-race is fixed during the test. Although the nominal rotation speed of the driving motor is 400 rpm. Its actual speed can hardly be kept constant due to oil pressure fluctuation. According to the geometric parameters of the bearing listed in Table 1, the nominal BCFs are calculated and given in Table 2. It is important to note that the BCFs are time-varying in the test since the bearings are not operating at a constant speed. To overcome this, those BCFs are normalized by the rotating frequency of outer race (in terms of order) and listed in Table 2.

**Figure 16.**
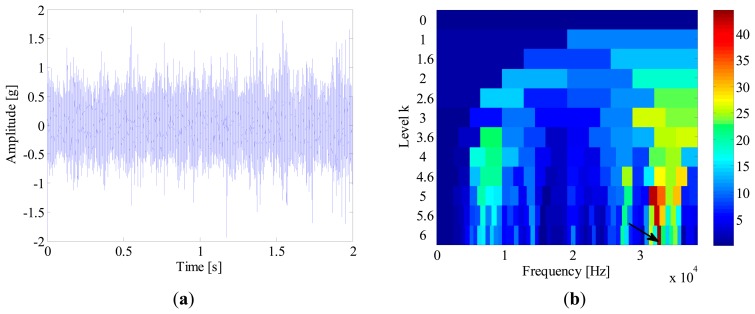
(**a**) Waveform and (**b**) kurtogram of the bearing signal.

A tri-axial accelerometer is mounted on the shaft end to collect the bearing vibration signals. The vibration signals are acquired for 2 s at a sampling rate of 76,800 Hz. Since the vibration in the vertical direction is constrained by the loading wheel, its amplitude is not as large as that in the horizontal. Therefore, the vibration signals in the horizontal direction are more informative and selected for further processing. In this experiment, the locomotive bearings are tested blindly and we don't know their fault types in advance. Figure 16a shows the waveform of a bearing vibration signal, from which no distinct impulses can be detected. To identify the bearing's health state, the SK-based method and IMF-AEOA are applied to the vibration signal, respectively.

**Figure 17.**
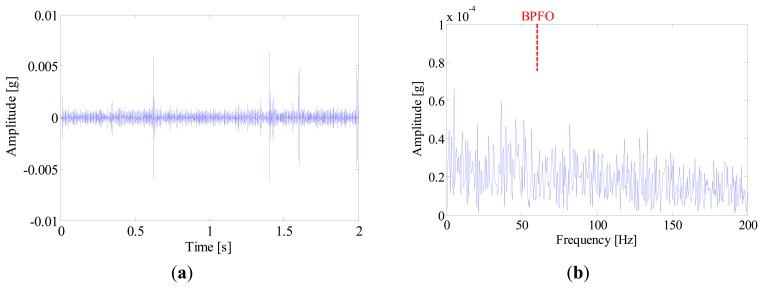
(**a**) Waveforms and (**b**) envelope spectrum of the band-pass filtered signal.

Figure 16b illustrates the kurtogram of the raw signal, from which the ‘optimal’ demodulation frequency-band can be obtained as 32,400∼33,000 Hz. Based on this information, the raw signal is band-pass filtered and the resultant waveform is presented in Figure 17a. It is noted that several high amplitude impulses with random time distribution are extracted from the filtered signal. However, those components come from the rubbing between the drive system and the chassis, rather than real faults. Therefore, when envelope analysis is applied to the filtered signal, no BCF can be detected in the envelope spectrum as given in Figure 17b. Therefore, the diagnostic result of SK-based approach is that the bearing is in a healthy state.

**Figure 18.**
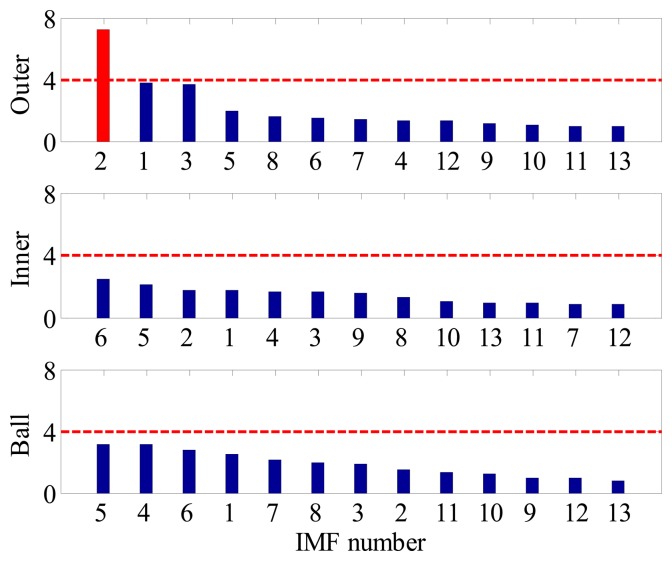
FSM of locomotive bearing signal.

**Figure 19.**
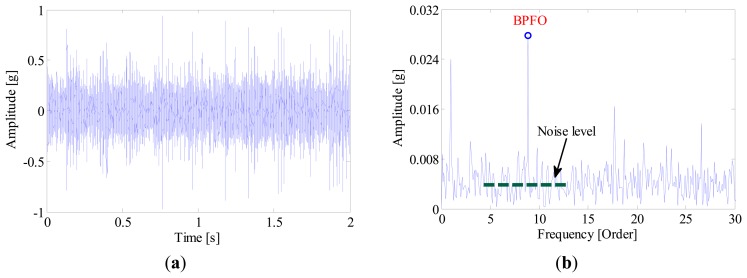
(**a**) Waveform and (**b**) envelope order spectrum of IMF2.

For comparison, the IMF-AEOA is performed on the same signal, and the FSM of the bearing vibration is illustrated in Figure 18. From this figure, it can be concluded that a defect may occur in the outer-race of the bearing. In addition, the FSM also reveals that the IMF2 is most sensitive to the outer-race fault. According to this, the waveforms of IMF2 are selected and presented in Figure 19a. It is found that the some sharp impulses caused by an outer-race fault become visible in the figure. By EOS, a dominant line locating at BPFO is recovered as shown in Figure 19b, which provides a clear evident for the outer-race fault.

**Figure 20.**
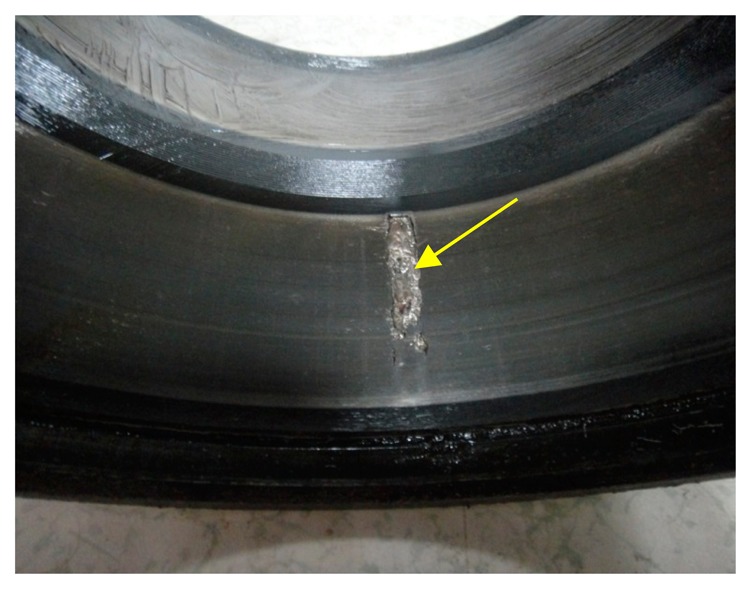
Outer-race of the dismantled bearing.

To validate the effectiveness of IMF-AEOA, the locomotive bearing is dismantled, and a picture of its outer-race is shown in Figure 20. From this figure, the spall fault in the outer-race can be seen obviously.

**Figure 21.**
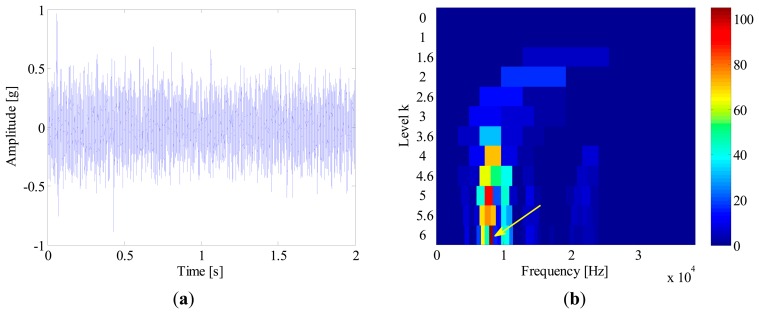
(**a**) Waveform and (**b**) kurtogram of the bearing signal.

**Figure 22.**
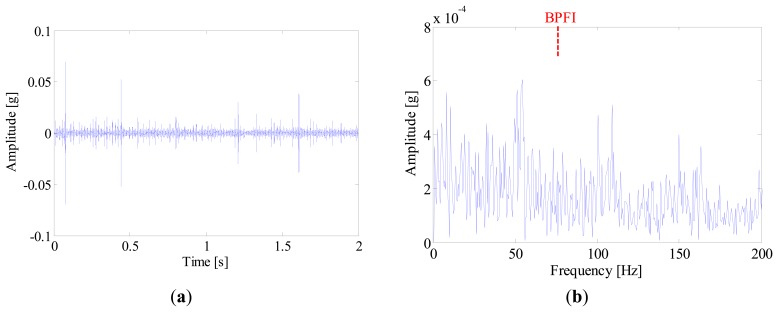
(**a**) Waveforms and (**b**) envelope spectrum of the band-pass filtered signal.

Besides external interference, the speed variation is also an obstacle that affects the diagnostic accuracy of locomotive bearings. For demonstration, Figures 21 and 22 illustrate the diagnosis of another locomotive bearing with an unknown fault based on the SK approach. As mentioned above, since the rotation speed of driving wheel (as well as the outer-race) cannot keep constant in the test, the smearing problem occurs in the envelope spectrum as shown in Figure 22b. From this figure none of the fault features can be identified.

**Figure 23.**
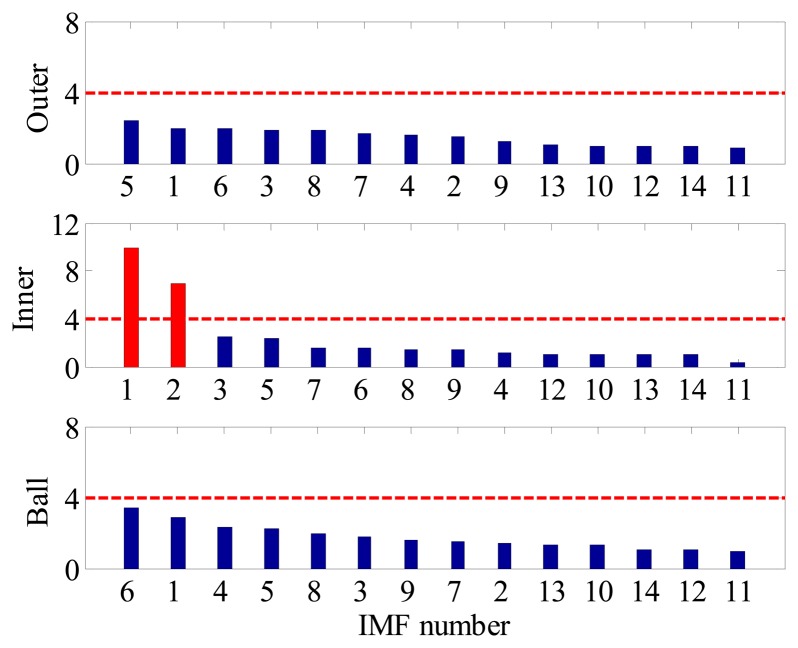
FSM of locomotive bearing signal.

**Figure 24.**
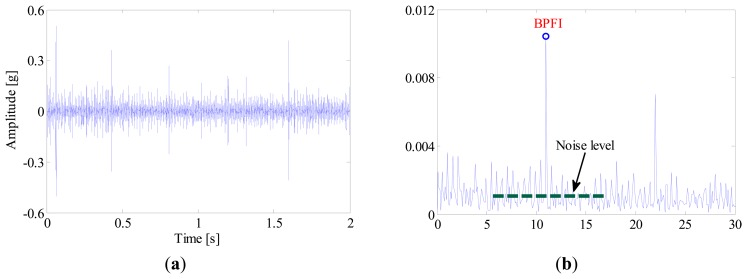
(**a**) Waveform and (**b**) envelope order spectrum of IMF1.

**Figure 25.**
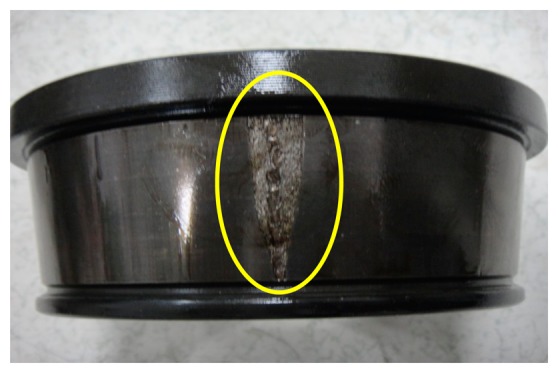
Inner-race of the dismantled bearing.

In the proposed method, the envelope of each IMF is resampled from the time domain to the angular domain, hence the influence of speed variation can be effectively removed. Figures 23 and 24 give the FSM and the most sensitive IMF component. By inspecting Figure 24b, one can conclude that the smearing problem is addressed in the EOS of IMF1, and the inner-race fault is extracted and identified even under non-stationary working conditions. Figure 25 shows the defect in the inner-race of the bearing.

**Figure 26.**
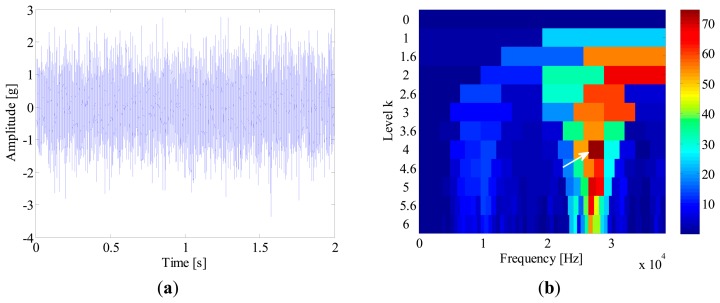
(**a**) Waveform and (**b**) kurtogram of the bearing signal.

**Figure 27.**
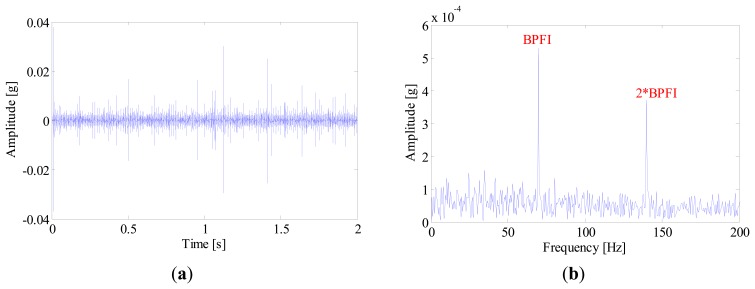
(**a**) Waveforms and (**b**) envelope spectrum of the band-pass filtered signal.

Some experimental investigations are also carried out to highlight the advantage of the proposed method in multi-fault detection. For instance, Figures 26 and 27 present the diagnostic procedure of a locomotive bearing using the SK approach. From the envelope spectrum shown in Figure 27b, one can observe the BPFI and its harmonics, and no other BCFs can be identified, so this signature indicates that a defect may occur in the inner-race.

**Figure 28.**
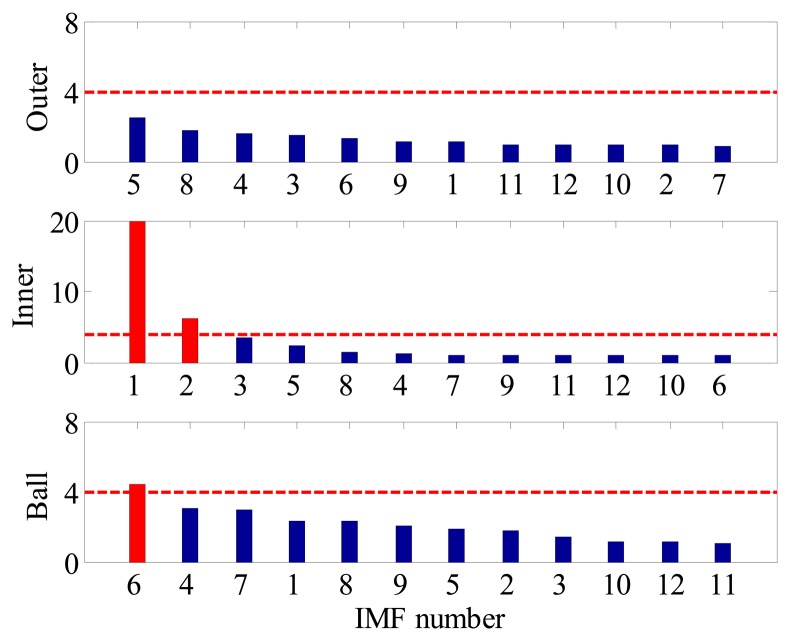
FSM of locomotive bearing signal.

**Figure 29.**
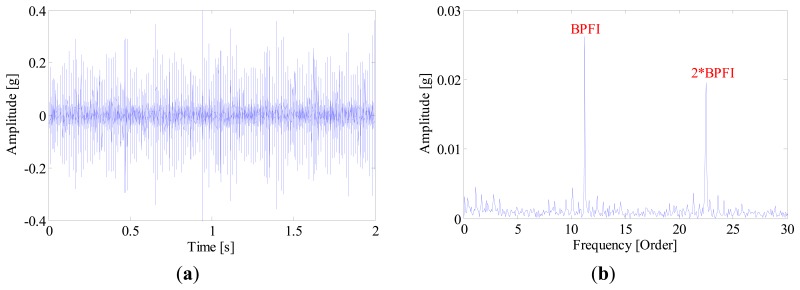
(**a**) Waveform and (**b**) envelope order spectrum of IMF1.

**Figure 30.**
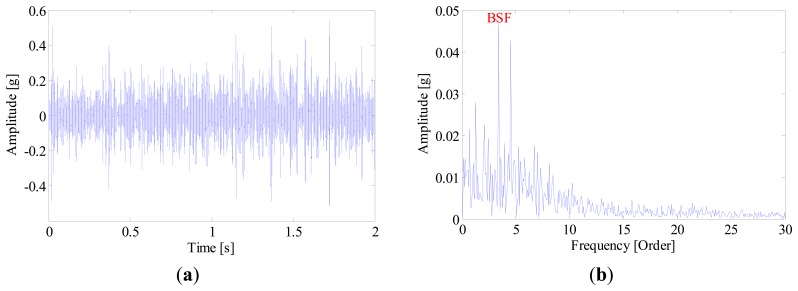
(**a**) Waveform and (**b**) envelope order spectrum of IMF6.

**Figure 31.**
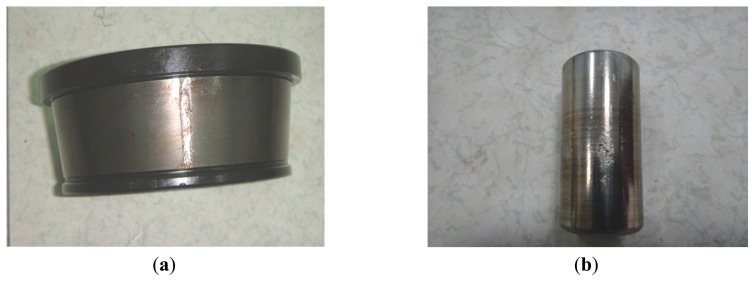
Inner-race and roller of the dismantled bearing.

For comparison, the same vibration signal is processed by the proposed method, which is illustrated in Figures 28, 29 and 30. However, the FSM gives a different diagnostic conclusion, *i.e.*, both inner-race and ball faults occur in the bearing. It also reveals that the fault signatures of the inner-race and ball fault are contained in IMF1 and IMF6, respectively. To further validate its effectiveness, the locomotive bearing is disassembled. As expected, both inner-race and roller faults were found, as shown in Figure 31.

## 6. Conclusions

The vibration signals of rolling element bearings used in the industrial field usually suffer from multiple vibration sources, low signal to noise ratios and time-varying statistical parameters, which cause difficulties in current diagnostic approaches. To establish a more reliable diagnostic method for bearings under such harsh working conditions, an IMF-based adaptive envelope order analysis technique is proposed in this paper. Some conclusions can be drawn as follows:

EEMD is an effective signal processing tool which could adaptively decompose the raw bearing signal into IMFs with different frequency bands. By using EEMD, the fault signature will be enhanced in certain IMF components. However, it is noted that the fault impulses can hardly be detected in IMF components in the presence of heavy noise and random impulsive interferences. Moreover, due to speed variation, those impulses are not equally spaced in time domain, which in turn leads to smearing problems in the spectrum. To remove the spectral smearing effect caused by speed variation, the envelope order analysis is further employed to transform the envelope of each IMF from the time domain to the angular domain. In this procedure, the BCFs of real faults are enhanced in the envelope order spectrum due to its angular cyclo-stationary characteristics, while strong interferences are eliminated since they are randomly distributed in the vibration signal. To select the optimal IMF containing the richest diagnostic information for decision making, a fault sensitive matrix is finally established in this work, which overcomes the limitation of maximum kurtosis based methods. By combining the advantages of EEMD, envelope order tracking and IMF sensitive analysis, both single and multi-faults of bearing can be identified successfully even under varying speed operations and strong interferences.

## Figures and Tables

**Table 1.** Geometric parameters of the roller bearing.

**Pitch Diameter (mm)**	**Roller Diameter (mm)**	**Contact Angle (degree)**	**Number of Rollers**
180	23.775	9	20

**Table 2.** BCFs of the bearing.

**Items**	**BCFs in Hz**	**BCFs in Order**
BPFO	57.97	8.695
BPFI	75.37	11.305
BSF	24.81	3.721
